# MUC4 stabilizes HER2 expression and maintains the cancer stem cell population in ovarian cancer cells

**DOI:** 10.1186/1757-2215-4-7

**Published:** 2011-04-26

**Authors:** Moorthy P Ponnusamy, Parthasarathy Seshacharyulu, ArokiaPriyanka Vaz, Parama Dey, Surinder K Batra

**Affiliations:** 1 Department of Biochemistry and Molecular Biology, University of Nebraska Medical Center, Omaha, NE 68198-5870, USA; 2 Eppley Institute for Research in Cancer and Allied Diseases, University of Nebraska Medical Center, Omaha, NE 68198-5870, USA

**Keywords:** MUC4, HER2, CD133, Side Population, Cancer Stem Cells, Ovarian Cancer

## Abstract

**Background:**

Recent evidence has suggested that the capability of cancer to grow, propagate and relapse after therapy is dependent on a small subset of the cell population within the tumor, called cancer stem cells. Therefore, this subpopulation of cells needs to be targeted with different approaches by identification of unique stem-cell specific target antigens. One of the well known tumor antigens is the epithelial cell mucin MUC4, which is aberrantly expressed in ovarian cancer as compared to the normal ovary and plays a pivotal role in the aggressiveness and metastasis of ovarian cancer cells. In the present study, we aimed to analyze the cancer stem cell population in MUC4 overexpressed ovarian cancer cells.

**Methods:**

MUC4 was ectopically overexpressed in SKOV3 ovarian cancer cells. Western blot analysis was performed for MUC4, HER2, CD133, ALDH1 and Shh expression in MUC4 overexpressed cells. Confocal analysis of MUC4, HER2 and CD133 was also done in the MUC4 overexpressed cells. CD133 and Hoechst33342 dye staining was used to analyze the cancer stem cell population via FACS method in SKOV3-MUC4 cells.

**Results:**

MUC4 overexpressed SKOV3 cells showed an increased expression of HER2 compared to control cells. MUC4 overexpression leads to increased (0.1%) side population (SP) and CD133-positive cancer stem cells compared to the control cells. Interestingly, the tumor sphere type circular colony formation was observed only in the MUC4 overexpressed ovarian cancer cells. Furthermore, the cancer stem cell marker CD133 was expressed along with MUC4 in the isolated circular colonies as analyzed by both confocal and western blot analysis. HER2 and cancer stem cell specific marker ALDH1 along with Shh, a self-renewal marker, showed increased expression in the isolated circular colonies compared to MUC4-transfected cells.

**Conclusion:**

These studies demonstrate that MUC4 overexpression leads to an enriched ovarian cancer stem cell population either directly or indirectly through HER2. In future, this study would be helpful for MUC4-directed therapy for the ovarian cancer stem cell population.

## Background

Ovarian cancer is a highly lethal disease which represents a great clinical challenge in gynecologic oncology. It is asymptomatic until the disease is in the late stage, causing it to have the highest fatality-to-case ratio of all gynecologic malignancies. There is emerging evidence showing that cancer stem cells are capable of regenerating tumors and they are responsible for the aggressiveness of the disease, metastasis and resistance to therapy [1]. Cancer stem cells, like somatic stem cells, are thought to be capable of self-renewal or unlimited proliferation. A recent study describes that ovarian cancer cell lines were shown to possess "side population" (SP) cells that have been described as cancer stem cells due to their ability to differentiate into tumors with different histologies, similar to the pluripotent character of stem cells [1]. It is now believed that cancer often relapses after the treatment due to the stem-like population in some solid tumors [2]. Although advanced ovarian cancer is generally initially responsive to standard chemotherapies (cisplatin and paclitaxel), it is almost inevitably followed by the drug resistant phenotype. One accepted hypothesis about chemoresistance is that standard therapies fail to target tumor progenitors, which are like normal stem cells, because of the expression of membrane efflux transporters [1].

The alterations in the mucin expression or glycosylation pattern is often associated with the development of cancer via influencing cellular growth, differentiation, transformation, adhesion, invasion and immunosuppression [3,4]. MUC4 frequently displays an altered expression under the pathological conditions of many cancers [3,4]. Previously, our study has revealed an aberrant expression of MUC4 mucin in > 90% of different histological subtypes and grades of ovarian tumors with very low or undetectable expression in the normal ovary [5]. Overexpression of MUC4 mRNA has also been reported in ovarian cancer [6]. In our previous study, we showed that MUC4 interacts and stabilizes HER2 in both ovarian and pancreatic cancer cells [7,8]. We have further shown that MUC4 induces the epithelial to mesenchymal transition (EMT) through the upregulation of N-cadherin, and thereby induces metastasis of human ovarian cancer cells [9]. A recent study has shown that HER2 amplification regulates the mammary stem/progenitor cell population and promotes carcinogenesis, tumorigenicity and invasive properties [10]. Recently, Engelmann et al have demonstrated that MUC1 (a membrane bound mucin) is also expressed in the mammary stem/progenitor cells [11] and is important in the future application of MUC1-based therapies for complete cancer eradication.

The aforementioned observations suggest that MUC4 may have an important role in the pathogenesis of ovarian cancer. In this study, we have investigated increased expression of HER2 and the cancer stem cell population in MUC4 overexpressed ovarian cancer cells. Further, we have analyzed cancer stem cell and self-renewal specific markers in the isolated populations. These studies indicate that MUC4 induces HER2 expression and may enrich the cancer stem cell population in ovarian cancer stem cells.

## Methods

### Generation of MUC4 construct

We generated a MUC4 minigene construct to overcome the transfection associated problems due to its large size and to investigate the biological function and effect of MUC4 expression in OC cells [8,12]. The resultant MUC4 cDNA was sub-cloned into the pSecTag-C vector for further transfection studies.

### Cell culture and transfection procedure

SKOV3 cells was procured from ATCC (Manassas, VA, U.S.A.) and cultured in DMEM supplemented with 10% fetal calf serum and antibiotics. The MUC4 gene construct along with the empty vector control pSecTag-C, were transfected in SKOV3 OC cells by Fugene (Invitrogen) following the manufacturer's protocol. Transfected cells were selected in the medium containing 200 μg/ml zeocin (30 Days) and the drug-resistance (zeo+) clones (three from the empty vector, five from the MUC4 gene construct transfected) were selected from different plates and studied after expansion [8,9].

### Immunoblot assay

SKOV3 derived cell lines were processed for protein extraction and Western blotting using standard procedures. Briefly, the cells were washed twice in PBS and lysate was prepared in RIPA buffer (100 mM Tris, 5 mM EDTA, 5% NP40; pH8.0) containing protease inhibitors (1 mM phenyl-methyl sulphonyl fluoride, 1 μg/ml aprotinin, 1 μg/ml leupeptin). SDS-PAGE (10%) was performed under standard conditions. Resolved proteins were transferred on to the PVDF membrane. After quick washing in PBST (Phosphate buffered saline and 0.1% Tween 20), the membranes were blocked in 5% nonfat dry milk in PBS for at least 2 h and then incubated with primary antibodies MUC4 (mouse), HER2 (rabbit), ALDH1 (rabbit), CD133 (rabbit), Shh (rabbit) and β-actin (mouse) (diluted in 5% non fat dry milk in PBS) overnight at room temperature. Then the membranes were washed (3 × 10 min) in PBST at room temperature and probed with 1:2000 diluted secondary antibodies (anti-mouse and anti-rabbit) for 1 h at room temperature and washed 5 × 10 min with PBST. The signal was detected with an ECL chemiluminescence kit (Amersham Bioscience, UK).

### Confocal immunofluorescence microscopy

MUC4 and vector transfected SKOV3 cells were grown on sterilized cover slips for 20 h. Cells were washed with Hanks buffer containing 0.1 M HEPES, and fixed in ice-cold methanol at 20°C for two minutes and blocked with 10% goat serum (Jackson Immunoresearch Labs, Inc., West Grove, PA, U.S.A.) containing 0.05% Tween-20 for at least 30 minutes. For Phalloidin staining, cells were fixed with 3.6% formaldehyde-PBS solution and followed by permeabilization with 0.1% TritonX-100 in PBS for 20 min at room temperature. After the blocking step and a quick wash in PBS, cells were incubated with the MUC4, HER2 and CD133 for 60 minutes at room temperature. Then cells were washed (4 × 5 minutes each washing) with PBS containing 0.05% Tween-20 (PBS-T) and then incubated with FITC-conjugated anti-mouse (green) and Texas red conjugated anti-rabbit (red) secondary antibodies (Jackson Immunoresearch labs, Inc., West Grove, PA) for 30 minutes at room temperature in the dark. Propidium iodide was used for nuclear staining. Cells were washed (5 × 5 minutes) again and mounted on glass slides in anti-fade Vectashield mounting medium (Vector Laboratories, Burlingame, CA). Laser confocal microscopy was performed by using a LSM 510 microscope (Carl Zeiss GmbH, Germany). Microphotographs of different stainings were taken in different channels separately.

### Hoechst 33342 dye and CD133 staining for Flowcytometry analysis

Hoechst 33342 dye based FACS analysis have been used to determine the SP and NSP population in MUC4 overexpressed and control SKOV3 cells. Cells were stained with Hoechst 33342 (Sigma) as previously described [13]. SP cells actively pump-out the dye (Hoechst 33342) and hence exhibit low fluorescence as compared to the NSP cells. Briefly, single cell suspension of OC cells was prepared at a density of 2 × 10^6^/ml in pre-warmed DMEM mixed with Hoechst 33342 dye (5 μg/ml). The cells were incubated in water bath at 37°C for 60 minutes and subsequently spun and re-suspended in cold HBSS+ (Hanks Balanced Salt Solution) containing 2 μg/ml propidium iodide (PI) for dead cell discrimination. Finally, the samples were run directly on the FACS and counted according to the strength of staining. The Hoechst dye was excited with the UV laser at 350 nm and its fluorescence measured with a 450/20 BP filter (Hoechst blue) and a 675 EFLP optical filter (Hoechst red). For the CD133-FITC and FACS analysis, 2 × 10^7 ^cells were incubated with FcR blocking reagent (MACS, Miltenyi Biotech) for 30 minutes. Then it was incubated with surface marker antibody CD133-FITC (MACS, Miltenyi Biotech) for 10 minutes. Finally, FACS analysis was carried out to count CD133-positive populations in MUC4-transfected and control SKOV3 cells.

## Results

### Ectopic Expression of MUC4 in SKOV3-Ovarian Cancer cells

**Figure 1:**
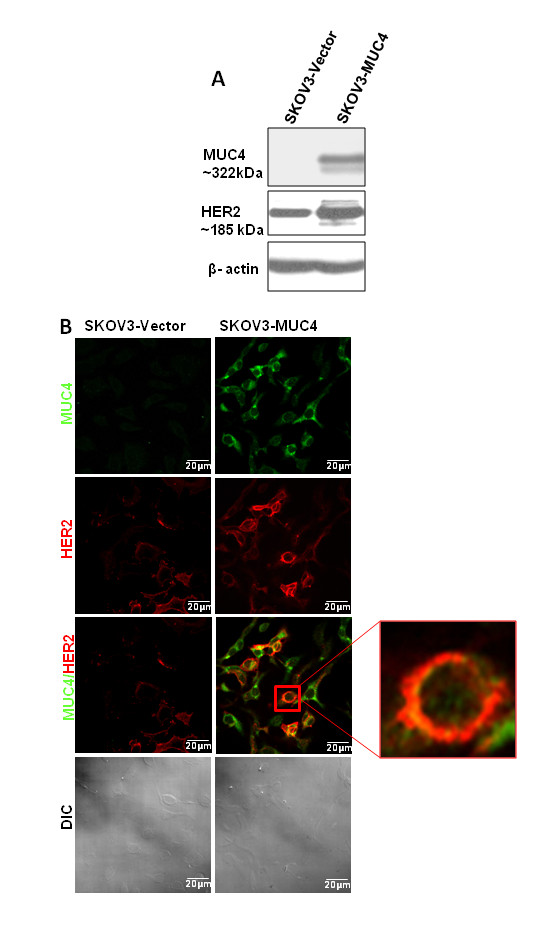
**Western blot and confocal analysis of MUC4 and HER2 in SKOV3 cells**. **(A) **Western blot analysis of MUC4 expression and its derived sub lines SKOV3 Vec (empty vector p-SecTaq) and SKOV3 MUC4. A total of 20 μg protein from cell extracts was resolved by electrophoresis on a 2% SDS-agarose gel for MUC4 and 10% SDS-PAGE for HER2, transferred to polyvinylidene difluoride membrane, and incubated with anti-MUC4 monoclonal antibody. The membrane was then probed with horseradish peroxidase-labeled goat anti-mouse immunoglobulin. The signal was detected using an electrochemiluminescence reagent kit. MUC4 mucin is a high molecular weight glycoprotein and the predicted size of the mini MUC4 protein is 320 kda. β-actin served as a loading control. **(B) **Localization of MUC4 and HER2 by confocal microscopy in both the derived cells. Cells were grown at low density on sterilized cover slips, washed, and fixed in ice-cold methanol at -20°C. After blocking in 10% goat serum, cells were incubated with the anti-MUC4 mouse monoclonal and anti-HER2 rabbit polyclonal antibodies, washed, and followed by secondary incubation with FITC-conjugated goat anti-mouse IgG and anti rabbit PI used for nuclear staining (Scale bar-20 μm).

The MUC4 construct developed in our laboratory is similar to the wild-type MUC4 with 10% repetitive domain size of its originally described allele [8,9,12]. Expression of MUC4 in stable cell transfectants were evaluated by Western blot analysis using a MUC4 antibody (8G7), which was developed in our laboratory that recognizes an epitope in the tandem repeat domain of MUC4. This antibody recognizes a MUC4 protein band of approximately 322 kDa specifically in the MUC4 gene transfected clone (SKOV3-MUC4) and not in the vector control (SKOV3-Vec) (Figure 1A). Expression and localization of MUC4 was further confirmed by immunofluorescence confocal microscopy. Ninety percent of the SKOV3-MUC4 transfected cells showed localization of MUC4 in both cytoplasm and membranes (Figure 1B) and an absence of MUC4 localization in vector-transfected SKOV3 cells (Figure 1B).

### Overexpression of MUC4 stabilizes HER2 in ovarian cancer cells

In our previous studies we have shown that MUC4 interacts with HER2 in ovarian cancer and pancreatic cancer cells [7,8]. In the present study we have analyzed the expression of HER2 in MUC4 overexpressed ovarian cancer cells. Our result showed that HER2 was upregulated in MUC4 overexpressed SKOV3 cells compared to the vector control (Figure 1A). Furthermore, we have analyzed MUC4 and HER2 localization in the same cells by confocal immunofuorescence analysis. MUC4 and HER2 were co-localized in MUC4 overexpressed cells, most of which show an EMT phenotype [9]. Interestingly, a few cells also showed a circular phenotype along with MUC4-HER2 co-localization (Figure 1B). This suggests that ovarian cancer cells consist of a heterozygous population of cells having different phenotype.

### Side population and Non-side population in MUC4 transfected SKOV3 cells

**Figure 2:**
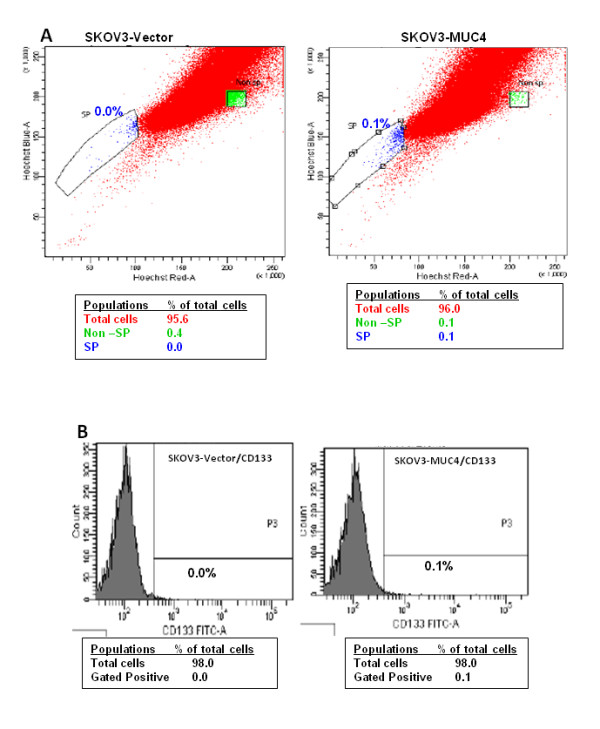
**Cancer stem cell population analysis in MUC4 overexpressed cells**. **(A) **Hoechest33342 dye analysis showed an increased (0.1%) side population (SP) in SKOV3-MUC4 cells compared to SKOV3-Vector cells. **(B) **FACS analysis of the CD133-positive population showed an enriched cancer stem cell population (0.1%) in MUC4-transfected SKOV3 cells compared to vector-transfected cells.

Recently, cancer stem cells have been identified as a minor population of cells sorted by flow cytometry based on their capacity to efflux the fluorescent DNA-binding dye Hoechst 33342. This is due to their overexpression of the ABCG2 drug resistance protein, one of the important characteristics of cancer stem/progenitor cells [14,15]. This population of cells was termed side population (SP) and the other population was called the non-side population (NSP). In the present study we have analyzed the number of SP and NSP cells in MUC4-transfected and control cells. Our results showed that MUC4-transfected SKOV3 cells were 0.1% more enriched in the SP population as compared to the vector control cells (Figure 2A). This suggests that overexpression of MUC4 leads to enrichment of the SP population in ovarian cancer cells.

### Increased number of CD133-positive cells in MUC4 overexpressed SKOV3 cells

CD133 is a cell surface antigen which was recognized as a stem cell specific marker for both normal and cancerous adult tissues. CD133 alone or along with other markers are currently used for the isolation of cancer stem cells from different cancer tissues and cell lines [16,17]. Qverexpressed MUC4 and control cells were stained with FITC-conjugated CD133 cells to analyze the percentage of the cancer stem cell population. Interestingly, MUC4-transfected SKOV3 cells showed a 0.1% increased CD133 population as compared to the control cells (Figure 2B). This further suggests that overexpression of MUC4 results in the enrichment of the cancer stem cell population in ovarian cancer cells.

### Circular colony formation in MUC4-transfected SKOV3 cells

**Figure 3:**
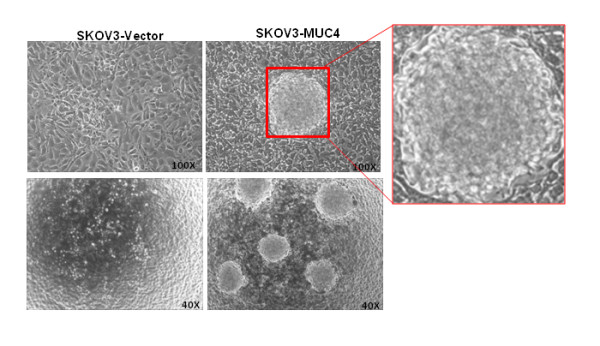
**Colony formation in MUC4-trasfected cells**. SKOV3-Vector and SKOV3-MUC4 cells were seeded in equal confluence and allowed to grow for up to 18 days. After the full confluence we observed a tumor sphere-like colony formation on the top of the cells. Circular colony formation was observed only in MUC4 overexpressed SKOV3 cells and no colonies were formed in SKOV3-vector cells. (Original magnification 100X upper panel and 40X lower panel).

Formation of spherical colonies has been reported to be a property characteristic of stem/progenitor cells and verifies a high developmental and proliferative potency of side population cells [11]. Interestingly, in our study we observed circular colony formation in MUC4-transfected SKOV3 cells when it became over confluent after three weeks (Figure 3). In contrast, no colony formation was observed in vector-transfected SKOV3 cells (Figure 3). We further isolated these colonies for the stem/progenitor marker analysis.

### Expression of cancer stem cell and self-renewal markers in circular colonies

**Figure 4:**
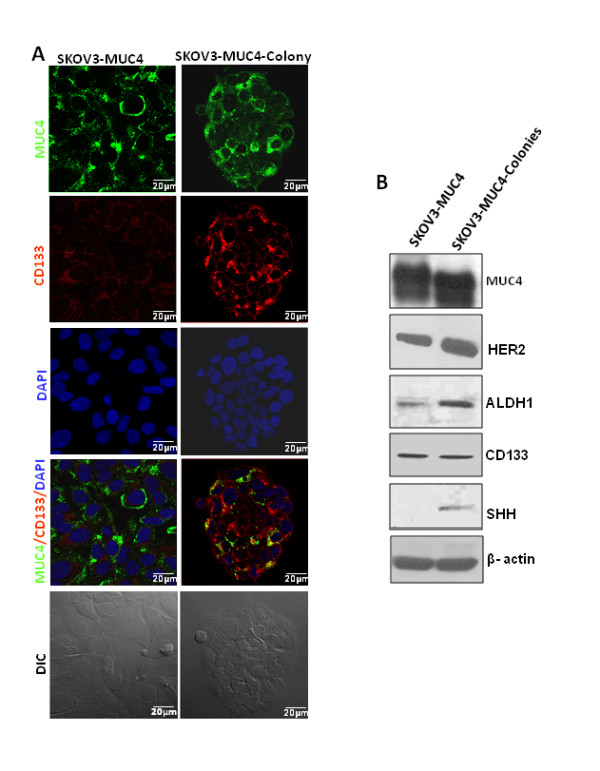
**Expression of cancer stem cell markers in circular colonies**. **(A) **Confocal analysis showed significant expression of CD133 (Red) in isolated colonies compared to SKOV3-MUC4 cells. MUC4 (Green) expression was seen in both isolated colonies and SKOV3-MUC4 cells. DAPI (Blue) was used as nuclear counter staining. **(B) **Western blot analysis showed MUC4, HER2, ALDH1, CD133 and Shh expression in SKOV3-MUC4 and isolated colonies from MUC4 overexpressed SKOV3 cells. β-actin served as a loading control. DIC - differential interference contrast and staining (Scale bar-20 μm).

**Figure 5:**
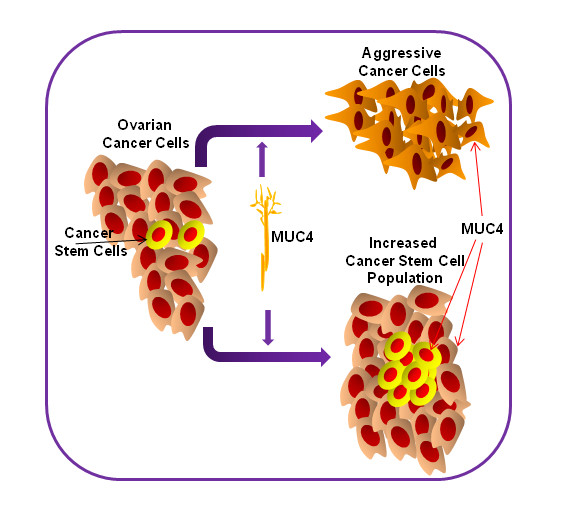
**Schematic representation showed that MUC4 overexpressed ovarian cancer cells induce the aggressiveness of the cancer cells and enrich the cancer stem cell population**. It also showed that MUC4 is expressed in the ovarian cancer stem cell population.

Cancer stem cells express numerous universal markers such as CD133, CD44, CD24, ESA and ALDH1 in different cancers [1]. Few of these markers were used for the confirmation of an MUC4 enriched cancer stem cell population. Stem cells and cancer stem cells are known to possess the phenomenal property of self-renewal which is maintained by few specific pathways such as Shh, Wnt and Notch [1]. The circular colonies or tumor spheres from MUC4-transfected SKOV3 cells were isolated and grown in a separate glass cover slip for the cancer stem cell marker analysis by confocal microscopy. The confocal results showed immunofluorescence staining of CD133 marker expression (Red) in the isolated colonies and SKOV3-MUC4 cells (Figure 4A). On the other hand, MUC4 (green) immunofluorescence staining is almost equal in both isolated colonies and SKOV3-MUC4 cells (Figure 4A). In our study we have also analyzed MUC4, HER2, ALDH1 and CD133 for the cancer stem cells and Shh for the self-renewal pathway in the isolated colonies from MUC4 overexpressed SKOV3 cells and SKOV3-MUC4. MUC4 expression was observed at an almost equal level in both SKOV3-MUC4 and isolated colonies although there was a minor molecular weight change in isolated colonies (Figure 4B). Interestingly, increased expression of HER2 was seen in isolated colonies compared to SKOV3-MUC4 cells. Expression of CD133 was also shown in both SKOV3-MUC4 and isolated colonies, whereas ALDH1 showed an increased expression in isolated colonies compared to MUC4 overexpressed SKOV3 cells (Figure 4B). In addition, the Shh self-renewal protein expression was observed only in isolated colonies, while there was no expression in SKOV3-MUC4 (Figure 4B). This suggests that the isolated colonies from MUC4 overexpressed cells behave like cancer stem cells which are capable of maintaining the self-renewal property (Figure 5).

## Discussion

Ovarian cancer is the fourth leading cause of cancer deaths among all women and has the highest mortality among the gynecologic cancers. It is one of the most challenging of all cancers to fight as it often goes undiagnosed until it has already advanced and metastasized. Furthermore, current therapeutic strategies have been inefficient and tumor recurrence is observed in up to 70% of patients with advanced stage ovarian cancer, even after treatment [18-20]. Numerous evidences have revealed that a small population of cells behaving like stem-cells are responsible for the tumor recurrence and disease aggressiveness [1]. These populations of cells known as cancer stem cells have been demonstrated to have roles in many cancers, such as cancers of the hematopoietic system, ovarian, breast, brain, prostate, pancreas, colon and liver [1,2,11,13,16,21-25]. To date, very few specific tumor antigens have been identified to target the cancer stem cell population to prevent tumor recurrence. Hence, the present study showed that MUC4 overexpression is enriching the cancer stem cell population and it is expressed in stem/progenitor cells in ovarian cancer cells.

MUC4 is known to be overexpressed in many types of carcinomas, including ovarian carcinoma [3,5,26]. The primary role of MUC4 is to protect the epithelial surface from injuries under normal physiological conditions, but overexpression is often correlated with the malignant phenotype. Our previous study reveals that MUC4 is aberrantly expressed in ovarian tumors [5] and in pancreatic tumors [3]. Another study from our laboratory shows that down-regulation of MUC4 is involved in the suppression of pancreatic tumor cell growth and metastasis [27]. In our recent study, we have shown a direct association of the MUC4 mucin with the metastatic human ovarian cancer phenotype and also provided experimental evidence for a functional role of MUC4 in altered growth behavioral properties of the tumor cell [8]. The ectopic expressed MUC4 gene has all the basic elements of the MUC4 gene but its tandem repeat region is only 10% of the wild-type MUC4 allele [8,12]. As predicted, MUC4 overexpressed cells expressed an approximately 322 kDa protein. The localization of the MUC4 protein was observed in both the membrane and cytoplasm of SKOV3 ovarian cancer cells.

Our previous study demonstrates an altered expression of the human epidermal growth factor receptor 2 (HER2), also known as ErbB2, in MUC4 overexpressing cells [8]. HER2 belongs to the epidermal growth factor receptor (EGFR) family. Most membrane-bound mucins have juxtamembrane domains with homology to the members of the EGF family [4]. MUC4 has been shown to act as an unorthodox ligand for ErBB2/HER2 [28], potentiating its responsiveness in cancer signaling [7,8]. Our recent finding showed that an overexpression of MUC4 increases the expression of HER2 in ovarian cancer cells [8] and stabilizes the HER2 oncoprotein in pancreatic cancer cells [7]. In the present study, we analyzed the expression of the HER2 protein in MUC4-transfected ovarian cancer cells. The exogenous MUC4 expression in ovarian cancer cells showed an increase in HER2 expression and colocalization, suggesting that MUC4 is involved in the stabilization of HER2 protein.

There is emerging evidence that small subpopulations that behave like stem cells are present in many types of cancer. These subpopulations are responsible for the initiation, drug resistance and tumor recurrence [1]. Korkaya and colleagues showed that HER2-overexpression in breast cancer cells enriched the cancer stem cell population, evidenced by an increased number of tumor sphere formation and cancer stem cell marker aldehyde dehydrogenase expression [10]. They have further shown an increased expression of stem cell regulatory genes, increased invasion *in vitro *and increased tumorigenesis in NOD/SCID mice [10]. This suggests that, in addition to genetic alterations such as amplification, the expression of the HER2 level is important for the maintenance of CSCs. Recently, a minor population of cells was isolated by flow cytometry based on their capacity to efflux the fluorescent DNA-binding dye Hoechst 33342 to identify cancer stem cells. This is one of the important properties for cancer stem/progenitor cells [14,15] because of the expression of ABCG2 drug resistance protein. In our study, we have observed enriched population of CSCs in MUC4-transfected ovarian cancer cells using Hoechst33342 dye and CD133-positive population analysis. Further, an interesting observation of tumor sphere like circular colony formation was observed predominantly in MUC4-transfected ovarian cancer cells and these colonies were isolated from the cells and analyzed with cancer stem cell markers. There was a slight change in the molecular weight of MUC4 protein in the isolated colonies suggesting that there may be some variation in glycosylation pattern, which will be explored in future studies (Figure 4B). The enriched cancer stem cell population in MUC4 overexpressed cells may be due to the increased expression of HER2 expression. Furthermore, isolated circular colonies showed significant expression of cancer stem cell specific markers CD133 and ALDH1 and the self-renewal maintenance marker Shh, which concludes that MUC4 is enriching the cancer stem cell population in ovarian cancer cells. Similarly, a recent study showed that a membrane-bound mucin MUC1 maintains a small population of stem/progenitor cells in the breast cancer (MCF7) cell line [11]. The fact that the vast majority of MCF7 stem cell enriched SP cells express MUC1 suggests that epithelial cancer stem cells would also be the targets of various immunotherapy approaches based on the MUC1 tumor antigen that has been designed with mature tumor cells in mind [11].

In conclusion, overexpression of MUC4 induces the HER2 level and enrichment of cancer stem cells in MUC4-transfected ovarian cancer cells. Circular colonies isolated from MUC4 overexpressed ovarian cancer cells show an increased expression of cancer stem cell specific markers (ALDH1and CD133) and the self-renewal maker (Shh). This suggests that MUC4 stabilizes HER2 and enriches the cancer stem cell population, by either a direct or indirect mechanism which is yet to be explored. Our study proves that MUC4 is not only expressed on mature cancer cells, but also on tumor cells that have multiple characteristics of stem/progenitor cells (Figure 5). This will help for the future application of a specific therapeutic target for cancer stem cells.

## Abbreviations

SP: Side Population; NSP: Non-Side Population; CSCs: Cancer stem cells; EMT: Epithelial to mesenchymal transition; EGFR: Epidermal Growth Factor Receptor.

## Competing interests

The authors declare that they have no competing interests.

## Authors' contributions

All the authors in this manuscript have read and approve the final manuscript. PPM: Conception and design, collection and/or assembly of data, data analysis and interpretation, manuscript writing. PS.: Experimental help and data analysis. AV: Data analysis and manuscript writing. PD.: Experimental help and manuscript writing. SKB: Conception and design, financial support, data analysis and interpretation, manuscript writing, and final approval of manuscript.

## References

[B1] Ponnusamy MP Batra SK Ovarian cancer: emerging concept on cancer stem cells J Ovarian Res 2008 1 4 10.1186/1757-2215-1-4 19014671 PMC2584054

[B2] Dean M Fojo T Bates S Tumour stem cells and drug resistance Nat Rev Cancer 2005 5 275 284 10.1038/nrc1590 15803154

[B3] Andrianifahanana M Moniaux N Schmied BM Ringel J Friess H Hollingsworth MA Buchler MW Aubert JP Batra SK Mucin (MUC) gene expression in human pancreatic adenocarcinoma and chronic pancreatitis: a potential role of MUC4 as a tumor marker of diagnostic significance Clin Cancer Res 2001 7 4033 4040 11751498

[B4] Hollingsworth MA Swanson BJ Mucins in cancer: protection and control of the cell surface Nat Rev Cancer 2004 4 45 60 10.1038/nrc1251 14681689

[B5] Chauhan SC Singh AP Ruiz F Johansson SL Jain M Smith LM Moniaux N Batra SK Aberrant expression of MUC4 in ovarian carcinoma: diagnostic significance alone and in combination with MUC1 and MUC16 (CA125) Mod Pathol 2006 19 1386 1394 10.1038/modpathol.3800646 16880776

[B6] Giuntoli RL Rodriguez GC Whitaker RS Dodge R Voynow JA Mucin gene expression in ovarian cancers Cancer Res 1998 58 5546 5550 9850092

[B7] Chaturvedi P Singh AP Chakraborty S Chauhan SC Bafna S Meza JL Singh PK Hollingsworth MA Mehta PP Batra SK MUC4 mucin interacts with and stabilizes the HER2 oncoprotein in human pancreatic cancer cells Cancer Res 2008 68 2065 2070 10.1158/0008-5472.CAN-07-6041 18381409 PMC2835497

[B8] Ponnusamy MP Singh AP Jain M Chakraborty S Moniaux N Batra SK MUC4 activates HER2 signalling and enhances the motility of human ovarian cancer cells Br J Cancer 2008 99 520 526 10.1038/sj.bjc.6604517 18665193 PMC2527793

[B9] Ponnusamy MP Lakshmanan I Jain M Das S Chakraborty S Dey P Batra SK MUC4 mucin-induced epithelial to mesenchymal transition: a novel mechanism for metastasis of human ovarian cancer cells Oncogene 2010 29 5741 5754 10.1038/onc.2010.309 20697346 PMC3005772

[B10] Korkaya H Paulson A Iovino F Wicha MS HER2 regulates the mammary stem/progenitor cell population driving tumorigenesis and invasion Oncogene 2008 27 6120 6130 10.1038/onc.2008.207 18591932 PMC2602947

[B11] Engelmann K Shen H Finn OJ MCF7 side population cells with characteristics of cancer stem/progenitor cells express the tumor antigen MUC1 Cancer Res 2008 68 2419 2426 10.1158/0008-5472.CAN-07-2249 18381450

[B12] Moniaux N Chaturvedi P Varshney GC Meza JL Rodriguez-Sierra JF Aubert JP Batra SK Human MUC4 mucin induces ultra-structural changes and tumorigenicity in pancreatic cancer cells Br J Cancer 2007 97 345 357 10.1038/sj.bjc.6603868 17595659 PMC2360313

[B13] Szotek PP Pieretti-Vanmarcke R Masiakos PT Dinulescu DM Connolly D Foster R Dombkowski D Preffer F Maclaughlin DT Donahoe PK Ovarian cancer side population defines cells with stem cell-like characteristics and Mullerian Inhibiting Substance responsiveness Proc Natl Acad Sci USA 2006 103 11154 11159 10.1073/pnas.0603672103 16849428 PMC1544057

[B14] Bunting KD ABC transporters as phenotypic markers and functional regulators of stem cells Stem Cells 2002 20 11 20 10.1002/stem.200011 11796918

[B15] Kim M Turnquist H Jackson J Sgagias M Yan Y Gong M Dean M Sharp JG Cowan K The multidrug resistance transporter ABCG2 (breast cancer resistance protein 1) effluxes Hoechst 33342 and is overexpressed in hematopoietic stem cells Clin Cancer Res 2002 8 22 28 11801536

[B16] Ferrandina G Bonanno G Pierelli L Perillo A Procoli A Mariotti A Corallo M Martinelli E Rutella S Paglia A Zannoni G Mancuso S Scambia G Expression of CD133-1 and CD133-2 in ovarian cancer Int J Gynecol Cancer 2008 18 506 514 10.1111/j.1525-1438.2007.01056.x 17868344

[B17] Ma S Lee TK Zheng BJ Chan KW Guan XY CD133+ HCC cancer stem cells confer chemoresistance by preferential expression of the Akt/PKB survival pathway Oncogene 2008 27 1749 1758 10.1038/sj.onc.1210811 17891174

[B18] Auersperg N Edelson MI Mok SC Johnson SW Hamilton TC The biology of ovarian cancer Semin Oncol 1998 25 281 304 9633841

[B19] Jemal A Siegel R Ward E Hao Y Xu J Thun MJ Cancer statistics, 2009 CA Cancer J Clin 2009 59 225 249 10.3322/caac.20006 19474385

[B20] Wong AS Auersperg N Ovarian surface epithelium: family history and early events in ovarian cancer Reprod Biol Endocrinol 2003 1 70 10.1186/1477-7827-1-70 14609432 PMC270003

[B21] Al-Hajj M Wicha MS ito-Hernandez A Morrison SJ Clarke MF Prospective identification of tumorigenic breast cancer cells Proc Natl Acad Sci USA 2003 100 3983 3988 10.1073/pnas.0530291100 12629218 PMC153034

[B22] Collins AT Berry PA Hyde C Stower MJ Maitland NJ Prospective identification of tumorigenic prostate cancer stem cells Cancer Res 2005 65 10946 10951 10.1158/0008-5472.CAN-05-2018 16322242

[B23] Dalerba P Dylla SJ Park IK Liu R Wang X Cho RW Hoey T Gurney A Huang EH Simeone DM Shelton AA Parmiani G Castelli C Clarke MF Phenotypic characterization of human colorectal cancer stem cells Proc Natl Acad Sci USA 2007 104 10158 10163 10.1073/pnas.0703478104 17548814 PMC1891215

[B24] Marsden CG Wright MJ Pochampally R Rowan BG Breast tumor-initiating cells isolated from patient core biopsies for study of hormone action Methods Mol Biol 2009 590 363 375 10.1007/978-1-60327-378-7_23 19763516

[B25] Mimeault M Hauke R Mehta PP Batra SK Recent advances in cancer stem/progenitor cell research: therapeutic implications for overcoming resistance to the most aggressive cancers J Cell Mol Med 2007 11 981 1011 10.1111/j.1582-4934.2007.00088.x 17979879 PMC4401269

[B26] Boman F Buisine MP Wacrenier A Querleu D Aubert JP Porchet N Mucin gene transcripts in benign and borderline mucinous tumours of the ovary: an in situ hybridization study J Pathol 2001 193 339 344 10.1002/1096-9896(2000)9999:9999<::AID-PATH798>3.0.CO;2-9 11241413

[B27] Singh AP Moniaux N Chauhan SC Meza JL Batra SK Inhibition of MUC4 expression suppresses pancreatic tumor cell growth and metastasis Cancer Res 2004 64 622 630 10.1158/0008-5472.CAN-03-2636 14744777

[B28] Ramsauer VP Pino V Farooq A Carothers Carraway CA Salas PJ Carraway KL Muc4-ErbB2 complex formation and signaling in polarized CACO-2 epithelial cells indicate that Muc4 acts as an unorthodox ligand for ErbB2 Mol Biol Cell 2006 17 2931 2941 10.1091/mbc.E05-09-0895 16624867 PMC1483030

